# Supplementary material for: Histologic-Based Tumor-Associated Immune Cells Status in Clear Cell Renal Cell Carcinoma Correlates with Gene Signatures Related to Cancer Immunity and Clinical Outcomes
Source: Biomedicines. 2022 Jan 29;10(2):323. doi: 10.3390/biomedicines10020323 (PMC8869140; doi:10.3390/biomedicines10020323)
Supplement: Supplementary file 1 [file biomedicines-10-00323-s001.zip › biomedicines-1528283-supplementary.pdf]

Supplementary Table S1. Clinicopathological characteristics of three-tier immunophenotype in 436 cases with localized ccRCCs in the principal cohort.

Variables	Desert	Excluded	Inflamed	P-value
Gender, n (%)				0.131
Female	72 (29.6)	17 (18.7)	28 (27.5)	
Male	171 (70.4)	74 (81.3)	74 (72.5)	
TNM stage, n (%)				0.004
I	193 (79.4)	64 (70.3)	63 (61.8)	
II	8 (3.3)	2 (2.2)	3 (2.9)	
III	42 (17.3)	25 (27.5)	34 (33.3)	
IV	0 (0.0)	0 (0.0)	2 (2.0)	
WHO/ISUP grade, n (%)				<0.001
1	47 (19.3)	5 (5.5)	8 (7.8)	
2	145 (59.7)	39 (42.9)	50 (49.0)	
3	46 (18.9)	40 (44.0)	28 (27.5)	
4	5 (2.1)	7 (7.7)	16 (15.7)	
Necrosis, n (%)				<0.001
Absent	229 (94.2)	65 (71.4)	76 (74.5)	
Present	14 (5.8)	26 (28.6)	26 (25.5)	
Sarcomatoid/rhabdoid components, n (%)				<0.001
Absent	240 (98.8)	87 (95.6)	89 (87.3)	
Present	3 (1.2)	4 (4.4)	13 (12.7)	
Recurrence, n (%)				<0.001
	230 (94.7)	69 (75.8)	80 (78.4)	
	13 (5.3)	22 (24.2)	22 (21.6)	
Cancer-specific mortality, n (%)				0.045
	239 (98.4)	87 (95.6)	95 (93.1)	
	4 (1.6)	4 (4.4)	7 (6.9)	

Supplementary Table S2. Clinicopathological characteristics of three-tier immunophenotype in 162 cases with ccRCCs in the TCGA cohort.

Variables	Desert	Excluded	Inflamed	P-value
Gender, n (%)				0.846
Female	27 (36.0)	13 (40.6)	19 (34.5)	
Male	48 (64.0)	19 (59.4)	36 (65.5)	
TNM stage, n (%)				< 0.001
I	52 (69.3)	11 (34.4)	19 (34.5)	
II	6 (8.0)	2 (6.2)	7 (12.7)	
III	15 (20.0)	14 (43.8)	14 (25.5)	
IV	2 (2.7)	5 (15.6)	15 (27.3)	
WHO/ISUP grade, n (%)				< 0.001
1	14 (18.7)	0 (0.0)	4 (7.3)	
2	47 (62.7)	9 (28.1)	19 (34.5)	
3	11 (14.7)	18 (56.2)	20 (36.4)	
4	3 (4.0)	5 (15.6)	12 (21.8)	
Necrosis, n (%)				< 0.001
Absent	72 (96.0)	26 (81.2)	37 (67.3)	
Present	3 (4.0)	6 (18.8)	18 (32.7)	
Sarcomatoid/rhabdoid components, n (%)				0.003
Absent	74 (98.7)	29 (90.6)	45 (81.8)	
Present	1 (1.3)	3 (9.4)	10 (18.2)	
Overall mortality, n (%)				< 0.001
Alive	60 (80.0)	9 (28.1)	26 (47.3)	
Death	15 (20.0)	23 (71.9)	29 (52.7)	

Supplementary Table S3. Clinicopathological characteristics of four-tier immunophenotype in 436 cases with localized ccRCCs in the principal cohort.

Variables	Desert	Immunosuppressed	Excluded	Inflamed	P value
Gender, n (%)					0.478
Female	72 (29.6)	30 (23.6)	7 (20.0)	8 (25.8)	
Male	171 (70.4)	97 (76.4)	28 (80.0)	23 (74.2)	
TNM stage, n (%)					<0.001
I	193 (79.4)	87 (68.5)	23 (65.7)	17 (54.8)	
II	8 (3.3)	4 (3.1)	0 (0.0)	1 (3.2)	
III	42 (17.3)	36 (28.3)	12 (34.3)	11 (35.5)	
IV	0 (0.0)	0 (0.0)	0 (0.0)	2 (6.5)	
WHO/ISUP grade, n (%)					<0.001
1	47 (19.3)	12 (9.4)	1 (2.9)	0 (0.0)	
2	145 (59.7)	66 (52.0)	11 (31.4)	12 (38.7)	
3	46 (18.9)	42 (33.1)	18 (51.4)	8 (25.8)	
4	5 (2.1)	7 (5.5)	5 (14.3)	11 (35.5)	
Necrosis, n (%)					<0.001
Absent	229 (94.2)	105 (82.7)	19 (54.3)	17 (54.8)	
Present	14 (5.8)	22 (17.3)	16 (45.7)	14 (45.2)	
Sarcomatoid/rhabdoid components, n (%)					<0.001
Absent	240 (98.8)	122 (96.1)	32 (91.4)	22 (71.0)	
Present	3 (1.2)	5 (3.9)	3 (8.6)	9 (29.0)	
Recurrence, n (%)					<0.001
	230 (94.7)	103 (81.1)	23 (65.7)	23 (74.2)	
	13 (5.3)	24 (18.9)	12 (34.3)	8 (25.8)	
Cancer-specific mortality, n (%)					0.146
	239 (98.4)	120 (94.5)	33 (94.3)	29 (93.5)	
	4 (1.6)	7 (5.5)	2 (5.7)	2 (6.5)	

Supplementary Table S4. Clinicopathological characteristics of four-tier immunophenotype in 162 cases with ccRCCs in the TCGA cohort.

Variables	Desert	Immunosuppressed	Excluded	Inflamed	P value
Gender, n (%)					0.967
Female	27 (36.0)	25 (37.3)	1 (25.0)	6 (37.5)	
Male	48 (64.0)	42 (62.7)	3 (75.0)	10 (62.5)	
TNM stage, n (%)					<0.001
I	52 (69.3)	29 (43.3)	0 (0.0)	1 (6.2)	
II	6 (8.0)	8 (11.9)	0 (0.0)	1 (6.2)	
III	15 (20.0)	17 (25.4)	4 (100.0)	7 (43.8)	
IV	2 (2.7)	13 (19.4)	0 (0.0)	7 (43.8)	
WHO/ISUP grade, n (%)					<0.001
1	14 (18.7)	4 (6.0)	0 (0.0)	0 (0.0)	
2	47 (62.7)	24 (35.8)	0 (0.0)	4 (25.0)	
3	11 (14.7)	30 (44.8)	3 (75.0)	5 (31.2)	
4	3 (4.0)	9 (13.4)	1 (25.0)	7 (43.8)	
Necrosis, n (%)					<0.001
Absent	72 (96.0)	50 (74.6)	4 (100.0)	9 (56.2)	
Present	3 (4.0)	17 (25.4)	0 (0.0)	7 (43.8)	
Sarcomatoid/rhabdoid components, n (%)					<0.001
Absent	74 (98.7)	61 (91.0)	4 (100.0)	9 (56.2)	
Present	1 (1.3)	6 (9.0)	0 (0.0)	7 (43.8)	
Overall Survival, n (%)					<0.001
Alive	60 (80.0)	30 (44.8)	0 (0.0)	5 (31.2)	
Death	15 (20.0)	37 (55.2)	4 (100.0)	11 (68.8)	

Supplementary Table S5. Clinicopathological characteristics of inflammation score in 436 cases with localized ccRCCs in the principal cohort.

Variables	Score 0	Score 1	Score 2	P value
Gender, n (%)				0.333
Female	72 (29.6)	30 (23.6)	15 (22.7)	
Male	171 (70.4)	97 (76.4)	51 (77.3)	
TNM stage, n (%)				<0.001
I	193 (79.4)	87 (68.5)	40 (60.6)	
II	8 (3.3)	4 (3.1)	1 (1.5)	
III	42 (17.3)	36 (28.3)	23 (34.8)	
IV	0 (0.0)	0 (0.0)	2 (3.0)	
WHO/ISUP grade, n (%)				<0.001
1	47 (19.3)	12 (9.4)	1 (1.5)	
2	145 (59.7)	66 (52.0)	23 (34.8)	
3	46 (18.9)	42 (33.1)	26 (39.4)	
4	5 (2.1)	7 (5.5)	16 (24.2)	
Necrosis, n (%)				<0.001
Absent	229 (94.2)	105 (82.7)	36 (54.5)	
Present	14 (5.8)	22 (17.3)	30 (45.5)	
Sarcomatoid/rhabdoid components, n (%)				<0.001
Absent	240 (98.8)	122 (96.1)	54 (81.8)	
Present	3 (1.2)	5 (3.9)	12 (18.2)	
Recurrence, n (%)				<0.001
Absent	230 (94.7)	103 (81.1)	46 (69.7)	
Present	13 (5.3)	24 (18.9)	20 (30.3)	
Cancer-specific mortality, n (%)				0.069
Alive	239 (98.4)	120 (94.5)	62 (93.9)	
Death	4 (1.6)	7 (5.5)	4 (6.1)	

Supplementary Table S6. Clinicopathological characteristics of inflammation score in 162 cases with ccRCCs in the TCGA cohort.

Variables	Score 0	Score 1	Score 2	P value
Gender, n (%)				0.977
Female	27 (36.0)	25 (37.3)	7 (35.0)	
Male	48 (64.0)	42 (62.7)	13 (65.0)	
TNM stage, n (%)				<0.001
I	52 (69.3)	29 (43.3)	1 (5.0)	
II	6 (8.0)	8 (11.9)	1 (5.0)	
III	15 (20.0)	17 (25.4)	11 (55.0)	
IV	2 (2.7)	13 (19.4)	7 (35.0)	
WHO/ISUP grade, n (%)				<0.001
1	14 (18.7)	4 (6.0)	0 (0.0)	
2	47 (62.7)	24 (35.8)	4 (20.0)	
3	11 (14.7)	30 (44.8)	8 (40.0)	
4	3 (4.0)	9 (13.4)	8 (40.0)	
Necrosis, n (%)				<0.001
Absent	72 (96.0)	50 (74.6)	13 (65.0)	
Present	3 (4.0)	17 (25.4)	7 (35.0)	
Sarcomatoid/rhabdoid components, n (%)				<0.001
Absent	74 (98.7)	61 (91.0)	13 (65.0)	
Present	1 (1.3)	6 (9.0)	7 (35.0)	
Overall survival, n (%)				<0.001
Alive	60 (80.0)	30 (44.8)	5 (25.0)	
Death	15 (20.0)	37 (55.2)	15 (75.0)	
